# The Old Family Doctor

**Published:** 1891-04-18

**Authors:** 


### The Old Family Doctor.

While we are pluming ourselves, not unjustly, on the progress of medical science and the increasing recognition in the popular mind of the importance of hygiene, it is well, now and then, to look at the other side of the scientific ledger, and see what we have lost as well as what we have gained. We have gained in this generation the specialists in pathology and practice, the man who knows one disease supremely well, but is, perhaps, too apt to think that all other ailments are only symptoms of his pet complaint; but we have lost thereby a much more kindly and not less useful personage—the old family doctor.

Is this the doctor's fault? I think not. Doctors, like other commodities, are subject to the law of the market—the law of supply and demand. Moreover, doctors, being living, sentient commodities, endowed by nature with

> "A palate and a set of teeth,
> And other delicate appliances
> To aid digestion"—

being perhaps endowed also by fate with wives and children, will naturally ply their craft in the fashion most likely to satisfy the needs of these endowments. The public cries out for the specialist, offers him large rewards, while it grudges the general practitioner the most modest honorarium; and so the specialist comes. Whether the public is a gainer by its choice may be doubted; but it has only itself to blame. Few men will, unless forced by circumstances, follow that branch of the profession which gets least of either praise or pudding. And it is fast becoming apparent that the modern family doctor does not hold the important place in our esteem which formerly belonged to him. Whether it is that the public are arriving at the conviction that, in the case of the doctor as of the governess, one person cannot be master of a dozen subjects, or that the medical men themselves are forcing the idea on the public, the fact remains that in any serious affliction, that does not require summary treatment, people, almost as a matter of course, place themselves in the hands of a specialist. Our modern facilities for locomotion encourage this, and patients take long journeys to see a complete stranger in face of the fact that the family doctor, through longer acquaintance with them, has acquired (possibly without their knowledge or against their will) such an amount of familiarity with their habits and constitutional tendencies as is seldom arrived at in the hurried interview with a popular specialist.

There is a lack of logic in this conduct. It is the fashion of the age to protest against the treatment of symptoms; it is equally the fashion of the age to go to doctors who can, in their treatment, have nothing but symptoms to go upon. And so the specialist finds his excuse for his being. Specialism brings wealth and notoriety to the man who is clever enough to see how the wind of public favour blows, and who can blame him if he profits by the lack of common sense displayed by the laity.

It certainly is not desirable to return to one type of doctor in whom our forefathers believed—the Sangrado, who—as the novice at whist when in doubt plays trumps—when in doubt tried bleeding. But are we of this generation so much wiser than those who went before us? If phlebotomy is no longer our only resource, if we no longer trust our lives to the witch, the Druid, or the travelling leech of yet earlier days, some of us yet bow to the machinations of a Sequah, while hypnotism, little understood and dangerous as it is, bids fair to become the favourite sensational "cure" of the higher classes. Again, it is the law of the market—the demand for something new and strange met by the unfailing supply, for it seems certain that disciples will always be ready to answer the call of the masters of any new creed; and that aid to all healing, Faith, will not fail to lend her assistance to these latter-day prophets as it did to their predecessors of old. In earlier times the exponents of various systems knew well enough how to throw back the responsibility of any failure in their cure on the sufferer's own head; we of to-day are, on the other hand, inclined to repudiate, both for ourselves and for nature, any share in a failure, and practically require of our doctors that they shall be infallible. Like the coxswain of a University boat-race, they must take all the blame of failure, but divide the praise when success is gained. They must be able to suggest new remedies when we revolt against those first prescribed. They must be always at hand to come to us when wanted, and never evince impatience if, on being called at an unreasonable hour, the need for the visit is not apparent. They must possess what is termed an excellent "bedside manner," and be able to inspire us with confidence both in them and in ourselves. In return for this small list of qualifications what does the public offer to make the bread-winning of its medical advisers a satisfaction even from a professional point of view?

Practically, it presents him with unbounded want of confidence in his judgment, and with every impediment to surmount in the way of gaining a clear view of any case. If it is an admitted opinion that doctors are humbugs, the public on its side can fairly be charged with frequent want of straightforwardness; and it is often to blame when the tact originally possessed by a medical man degenerates into hypocrisy. It does not really feel any gratitude for too great a display of candour. Radcliffe found this out to his cost when he once gave vent to his genuine opinion to the Princess Anne. She had "the blues," but she didn't want to be told so, and so Radcliffe soon afterwards received his dismissal, which neither benefited the Princess nor himself.

The old family doctor of the times immediately behind us had a far better chance of fair treatment than has the ordinary practitioner at present. He was frequently not only the doctor, but also the family friend and adviser, and his scoldings for any imprudence, when even it was without the range of his profession, were sure to be taken in good part. What have we in place of him?

His successor is probably a young fellow fresh from his hospital training, embued with the latest scientific ideas, and over-flowing with new views for the treatment of disease. He settles in the quiet village. The people at the Hall employ a doctor from the nearest big town, and all those who can afford it follow their example, except in cases of trivial illness. He finds himself practically the doctor of the poor, and dependent on clubs, &c., for the larger amount of his income. His ambition is, therefore, rather to migrate where he gets greater scope for his talents. Supposing he cannot accomplish this, he assumes the aspect of a disappointed man, and accepts obscurity. The tendency of the London medical student, if he is sufficiently brilliant, is to attach himself to some hospital, and to enter into the whirl and hurry of private and hospital practice combined. If possible, too, he must unite with these some literary efforts, embodying the outcome of his own experience. Naturally, he finds little time for anything beyond the limits of his own profession. And even his inquiries into the nature and causes of disease are bounded by the strictest materialism. He saw no traces of a soul in his studies in the dissecting room, nor was its action shown by any lectures on physioloy he ever heard. And as he has chosen a lot which makes him no man's con fidant, save on matters relating to stomach and liver, he never becomes a really "all-round" man, who can guage the effect of the mind's action on the body.

There is, indeed, a frequent tendency in modern medical education to breed a veiled contempt for human nature and to ignore the existence of one that is divine. To alleviate the sufferings of humanity often seems, with some great lights of the profession, less an object than the advance of science in the investigation of disease or the opportunity of performing a neat and pretty operation. He who discovers a new bacillus is more honoured than he who checks an epidemic; and though the pathologist is a most necessary member of the profession, we cannot help feeling that he sometimes forgets in his studies in "nature's laboratory, disease" that the ultimate aim of his labours should be, not diagnosis, but cure. This is not the spirit nor the end of the teaching and work of the first great Physician of the Christian era, nor the spirit in which His disciples cured the multitudes in the name of their Divine Master. I look upon such a spirit, this other spirit, as the Nemesis arising from an overweening confidence in the infallibility of the human intellect. Now and then, amidst the vanguard of the advancing army of science, stands out as a bright light, some one man who consecrates the gifts and triumphs of his intellect to the good of his fellow creatures apart from the glory he may reap in the paths of fame. But oftener is self-sacrifice and Christian deed to be found in those walks of life where treads his humbler brother, the now often too little considered general practitioner.

